# Mitral Valve Repair for Mitral Regurgitation Associated with Antiphospholipid Syndrome: A Case Report

**DOI:** 10.70352/scrj.cr.25-0470

**Published:** 2025-11-29

**Authors:** Masaru Yoshikai, Hisashi Sato, Nagi Hayashi, Kouta Shimauchi, Naoyo Nishida

**Affiliations:** 1 Department of Cardiovascular Surgery, Shin-Koga Hospital, Kurume, Fukuoka, Japan; 2 Department of Pathology, Shin-Koga Hospital, Kurume, Fukuoka, Japan

**Keywords:** antiphospholipid syndrome, mitral regurgitation, mitral valve repair

## Abstract

**INTRODUCTION:**

Antiphospholipid syndrome (APS) is a multisystem autoimmune disorder of hypercoagulability, characterized by arterial and venous thrombosis, recurrent fetal losses, thrombocytopenia, and circulating antiphospholipid antibodies. Among valvular manifestations associated with APS, mitral regurgitation (MR) is the most common, followed by aortic regurgitation. Surgical interventions for APS-related valvular diseases carry high perioperative morbidity and mortality. There are no established guidelines regarding the surgical management of APS-related MR.

**CASE PRESENTATION:**

A 46-year-old man underwent mitral valve repair for severe MR. Histopathological examination of the vegetation on the mitral valve revealed nonbacterial thrombotic endocarditis, and in combination with laboratory findings, the MR was diagnosed as associated with APS. Leaflet thickening and fusion progressed postoperatively, leading to moderate-to-severe MR and mitral stenosis within 2 years.

**CONCLUSIONS:**

In cases where MR is the initial presentation of APS, preoperative diagnosis of APS can be challenging. Detailed, frame-by-frame transesophageal echocardiographic evaluation may aid in the preoperative identification of subtle valvular abnormalities suggestive of APS. When thrombocytopenia and a prolonged activated partial thromboplastin time (APTT) are identified during preoperative evaluation for valvular heart disease, tests for lupus anticoagulant, anticardiolipin antibody, and anti-β2 glycoprotein I antibody should be performed, and consultation with hematologists is recommended. Valve repair for MR associated with APS, which preserves valvular tissue that may become diseased in the future, is likely to result in poor outcomes.

## Abbreviations

APS
: antiphospholipid syndrome
APTT
: activated partial thromboplastin time
MR
: mitral regurgitation
NBTE
: nonbacterial thrombotic endocarditis
TEE
: transesophageal echocardiography
TTE
: transthoracic echocardiography

## INTRODUCTION

APS is a multisystem autoimmune disorder of hypercoagulability, characterized by arterial and venous thrombosis, recurrent fetal losses, thrombocytopenia, and circulating antiphospholipid antibodies. Among valvular manifestations associated with APS, MR is the most common, followed by aortic regurgitation.^1)^ Surgical interventions for APS-related valvular diseases carry high perioperative morbidity and mortality.^1–3)^ We present a case in which APS was not diagnosed preoperatively, and mitral valve repair was performed for severe MR. The patient subsequently developed recurrent MR and mitral stenosis postoperatively.

## CASE PRESENTATION

**Fig. 1:**
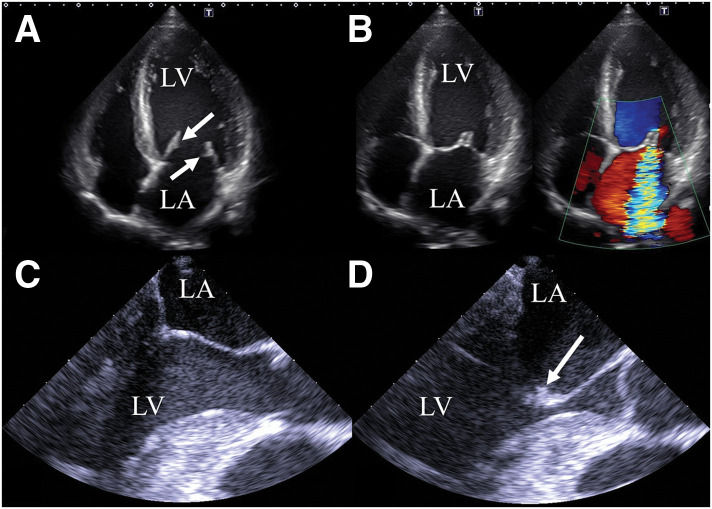
Transthoracic echocardiographic findings. Transthoracic echocardiography demonstrates mild leaflet thickening (arrows in [**A**]) and severe mitral regurgitation without leaflet prolapse (**B**). Transesophageal echocardiography reveals a nodular lesion on the tip of the anterior mitral leaflet (arrow in [**D**]), visible only during diastole (**C**, **D**). LA, left atrium; LV, left ventricle

**Fig. 2:**
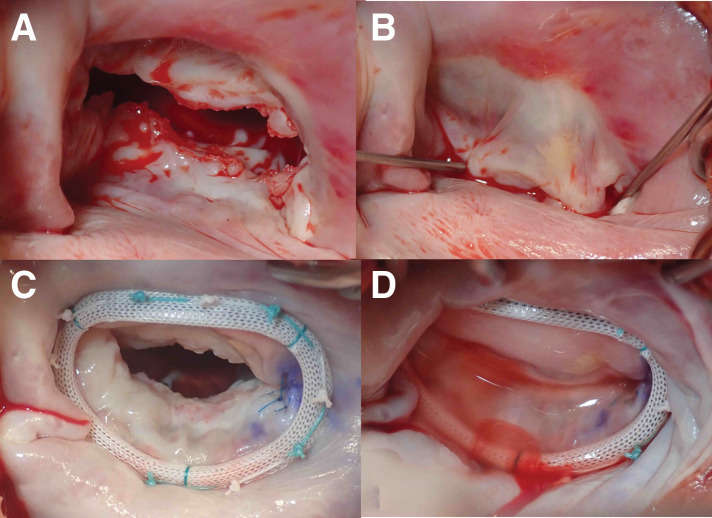
Operative findings. Operative findings reveal nodular lesions on the rough zone of both leaflets (**A**), sparing the clear zone (**B**). After completion of mitral valve repair, postoperative images demonstrate the excision of nodular lesions (**C**) and a well-established leaflet coaptation (**D**).

**Fig. 3:**
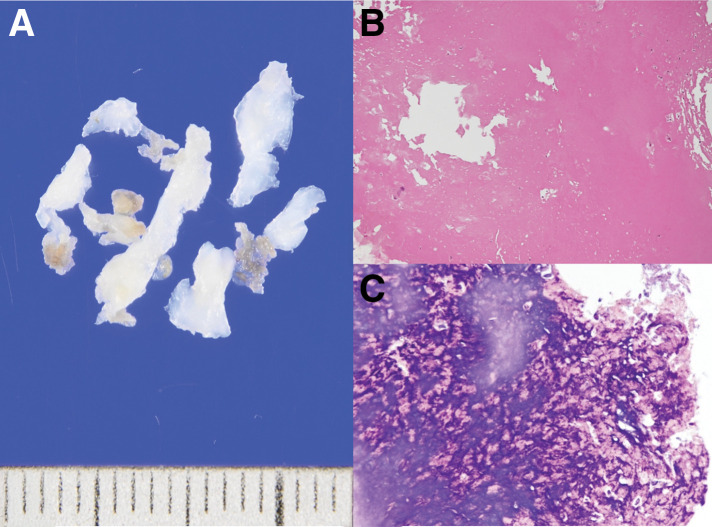
Gross appearance and histopathological examination. Gross appearance of the resected vegetations on the leaflets (**A**). Histopathological examination of the vegetation demonstrates a fibrin-rich thrombus (**B**: hematoxylin–eosin staining; **C**: phosphotungstic acid hematoxylin staining, in which fibrin is stained blue).

**Fig. 4:**
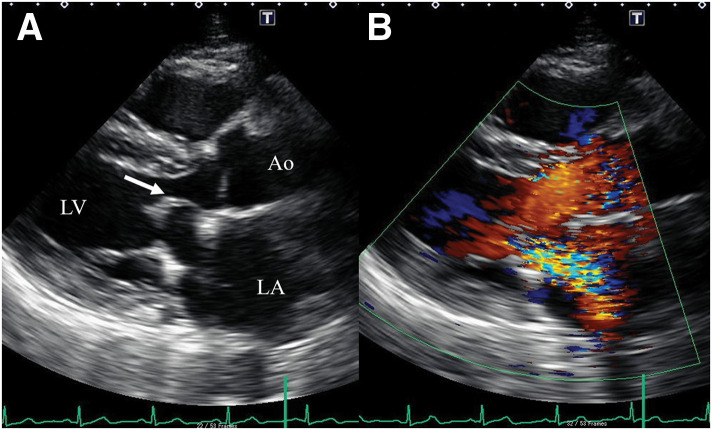
Transthoracic echocardiographic findings. Transthoracic echocardiography 2 years after valve repair shows thickening of both leaflets and doming of the anterior mitral leaflet (arrow in [**A**]), resulting in stenosis at the leaflet tips (**A**), along with moderate-to-severe mitral regurgitation without leaflet prolapse (**B**). Ao, aorta; LA, left atrium; LV, left ventricle

A 46-year-old man was referred for surgical treatment of severe MR. He had no history of arterial or venous thrombosis, or embolic events. Laboratory data revealed thrombocytopenia with a platelet count of 77000/μL, a mildly prolonged APTT (38.9 seconds; normal range, 24.3–36.0 seconds), and a slightly shortened prothrombin time (10.3 seconds; normal range, 10.5–13.5 seconds). On TTE, the mitral leaflets were mildly thickened (4 mm at the tips) but within normal limits (**Fig. 1A**), and no leaflet prolapse was noted (**Fig. 1B**). It also showed MR jet originating between P2 and P3, with a regurgitant fraction of 54% and regurgitant volume of 97.5 mL, consistent with severe MR (**Fig. 1B**). Mild mitral annular dilation was observed. The left ventricular end-diastolic diameter was 57 mm, and end-systolic diameter was 40 mm, with an ejection fraction of 58%. TEE revealed a 2-mm nodular lesion on the mitral leaflet visible only during diastole (**Fig. 1C** and **1D**), which had been overlooked preoperatively. Although he was asymptomatic, TTE revealed findings that indicated the need for surgical intervention. The surgery was performed via median sternotomy under cardiopulmonary bypass with cardioplegic arrest. Intraoperatively, verrucous lesions were observed on the rough zone of both mitral valve leaflets, whereas the clear zone was spared (**Fig. 2A** and **2B**) and were excised using a sharp curette. The cleft between the P2 and P3 scallop, which was the main source of regurgitation, was closed with direct sutures. A Carpentier-Edwards Physio II Annuloplasty Ring, 32-mm (Edwards Lifesciences, Irvine, CA, USA), was implanted (**Fig. 2C** and **2D**), resulting in the resolution of MR on TEE. The postoperative course was uneventful. Postoperative TTE demonstrated normalization of left ventricular dilatation, trivial MR, and a mean transvalvular pressure gradient of 2 mmHg. Histopathological examination of the excised lesions revealed fibrin-rich thrombus without evidence of microbial colonies, consistent with NBTE (**Fig. 3**). Later, laboratory testing showed positivity for lupus anticoagulant, anti-cardiolipin antibody, and anti-β2 glycoprotein I antibody, confirming a diagnosis of APS. Warfarin was discontinued 3 months after the surgery by the patient’s primary physician. Follow-up TTE at 1 year postoperatively showed mild-to-moderate MR and moderate mitral stenosis with mean transvalvular pressure gradient of 8 mmHg. At 2 years, TTE revealed progression to moderate-to-severe MR and mitral stenosis with thickened leaflets (**Fig. 4**). Reoperation is currently under consideration.

## DISCUSSION

Surgical treatment of APS-related valvular diseases carries high perioperative morbidity and mortality.^1–3)^ Therefore, establishing a diagnosis of APS prior to surgery is essential. However, as in the present case, MR can be the initial manifestation of APS, warranting careful clinical attention, and the detection of valvular lesions by cardiac imaging modalities remains challenging.^4)^ The pathogenesis of APS-related valve lesions involves the deposition of autoimmune complexes and complement, leading to valve thickening and the formation of fibrin-platelet thrombi (vegetation) on the valvular surface,^5)^ characteristic of NBTE,^6)^ making the identification of these findings by echocardiography crucial. Leaflet thickening typically occurs at the leaflet tips, specifically in the rough zone, and represents one of the earliest echocardiographic findings.^4,6)^ The diagnostic sensitivity of TTE for NBTE is low, at 45.2%, whereas TEE offers much higher sensitivity, of up to 97.1%.^4)^ TEE offers much higher diagnostic sensitivity for NBTE than TTE,^4)^ therefore, TEE should be performed when NBTE is suspected. In the present case, mild thickening of the rough zone was observed; however, the thickness measured 4 mm, which falls within the normal range. Moreover, TEE recorded a nodular lesion on the leaflet, seen in only 2 of 75 frames recorded at 31 frames per second. So, the lesion was overlooked preoperatively due to reliance on looped video review alone. This highlights the importance of thoroughly examining each frame when reviewing video loops, as even subtle lesions may be missed if frames are not carefully reviewed. In this case, the diagnosis of APS was also suggested by the preoperative laboratory findings; however, thrombocytopenia and prolongation of the APTT had been overlooked before surgery. Given the preoperative findings of thrombocytopenia and a prolonged APTT, assessment for lupus anticoagulant, anticardiolipin antibody, and anti-β2 glycoprotein I antibody, along with consultation with hematologists, should have been performed.

There are no established guidelines regarding the surgical management of APS-related MR, owing to the limited number of reported cases. In the present case, mild-to-moderate MR and moderate mitral stenosis were found 1 year after mitral valve repair, both of which had progressed to moderate-to-severe severity by the 2nd year. These findings are suggestive of recurrent and progressive NBTE, resulting in incomplete coaptation of the leaflets leading to MR, and leaflet thickening and fusion causing mitral stenosis. Although discontinuation of anticoagulation therapy (warfarin) by the patient’s primary physician may have contributed to the recurrence and progression of NBTE, the preservation of the mitral leaflets—the primary substrate for NBTE—during repair suggests an ongoing risk of disease reactivation.^7)^ In some cases, mitral valve repair may be technically challenging due to significant leaflet thickening and verrucous vegetations causing virulent tissue destruction.^2)^ Furthermore, in patients with APS secondary to systemic lupus erythematosus, steroid therapy itself may modify mitral valve pathology.^5)^ Mitral valve repair for APS-related MR is considered to reduce the risks associated with mechanical valve replacement, such as prosthetic valve thrombosis, thromboembolism, and bleeding, thereby potentially decreasing early postoperative mortality. However, because the valvular tissue, which may become diseased in the future, is preserved in mitral valve repair, this approach has significant limitations, and valve replacement is considered a more appropriate surgical option.^2)^ On the contrary, the choice of prosthetic valve is a significant issue when performing valve replacement. In the case of mechanical valves, the risk of thrombosis is higher, while bioprosthetic valves have been reported to fail prematurely.^8)^ In APS, long-term warfarin therapy is often required as a standard treatment. Given the prevalence of valve replacement in younger patients, mechanical valves have commonly been used,^1,3,4)^ however, their clinical outcomes have been poor. Colli et al., based on their experience with postoperative thromboembolic events—identified as the most frequent complication following mechanical valve replacement—shifted their perspective and proposed that bioprosthetic valves may represent a more suitable prosthetic option.^2)^ In mitral valve replacement for APS-related MR, regardless of whether a mechanical or bioprosthetic valve is used, neither can be considered the ideal prosthesis. When performing valve replacement, the choice of prosthetic valve remains controversial,^9)^ and further case accumulation should be required.

## CONCLUSIONS

In cases where MR is the initial presentation of APS, preoperative diagnosis of APS can be challenging. Detailed, frame-by-frame transesophageal echocardiographic evaluation may aid in the preoperative identification of subtle valvular abnormalities suggestive of APS. When thrombocytopenia and a prolonged APTT are identified during preoperative evaluation for valvular heart disease, tests for lupus anticoagulant, anticardiolipin antibody, and anti-β2 glycoprotein I antibody should be performed, and consultation with hematologists is recommended. Valve repair for MR associated with APS is likely to result in poor outcomes. When performing valve replacement, the choice of prosthetic valve remains controversial, and further case accumulation should be required for establishing guidelines regarding the surgical management of APS-related MR.

## DECLARATIONS

### Availability of data and materials

Data are available on request to the corresponding author.
